# *Peucedanum japonicum* Thunberg and Its Active Components Mitigate Oxidative Stress, Inflammation and Apoptosis after Urban Particulate Matter-Induced Ocular Surface Damage

**DOI:** 10.3390/antiox10111717

**Published:** 2021-10-28

**Authors:** Wan Seok Kang, Hakjoon Choi, Ki Hoon Lee, Eun Kim, Kyeong Jo Kim, Jin Seok Kim, Chang-Su Na, Sunoh Kim

**Affiliations:** 1 Central R&D Center, B&Tech Co., Ltd., Gwangju 61239, Korea; kws2602@hanmail.net (W.S.K.); ohchj12@naver.com (H.C.); leekh3261@daum.net (K.H.L.); rubsang84@gmail.com (E.K.); kkkjzzang@nate.com (K.J.K.); keki2000@naver.com (J.S.K.); 2 College of Korean Medicine, Dongshin University, Naju-si 58245, Korea; nakugi@hanmail.net

**Keywords:** urban particulate matter (UPM), *Peucedanum japonicum* Thunberg, corneal epithelial cells, wound healing, antioxidant, inflammation

## Abstract

We previously demonstrated that urban particulate matter (UPM) exposure decreases the migration activity and survival of human corneal epithelial cells (HCECs). Herein, we investigated the potential to improve the corneal wound-healing ability of *Peucedanum japonicum* Thunb. leaf extract (PJE) and its active components on UPM-induced ocular surface damage in vitro and in vivo. PJE effectively assisted wound healing without altering HCEC survival and enhanced catalase (CAT), heme oxygenase 1 (HO1) and glutathione peroxidase 1 (GPX1) antioxidant gene expression. A corneal wound was uniformly induced on the right eye in all experimental animals and divided into eight groups such as two control groups (wounded right eye group—NR and non-wounded left eye group—NL), UPM treated group and PJEs (25, 50, 100, 200, 400 mg/kg) treated groups. Corneal abrasion model rats exposed to UPM showed delayed wound healing compared to unexposed rats, but wound healing was dose-dependently enhanced by PJE oral administration. Seventy-two hours after wound generation, inflammatory cells, apoptotic cells and interleukin-6 (IL-6) expression were increased substantially after UPM exposure, but PJE treatment significantly reduced the wound to an almost normal level while enhancing re-epithelialization without changing corneal thickness. Next, we tried to identify the key molecules for enhancing wound healing through fractionation. The major compounds in the fraction, confirmed by high-performance liquid chromatography (HPLC), were chlorogenic acid (CA), neochlorogenic acid (NCA) and cryptochlorogenic acid (CCA). Each type of CA isomers showed slightly different half maximal effective (EC50) and maximal effective (ECmax) concentrations, and their mixtures synergistically enhanced HCEC migration. Thus, corneal abrasion wound recovery after UPM exposure improved after PJE treatment, and the active PJE components were identified, providing an important basis to develop therapeutics for ocular surface damage using PJE.

## 1. Introduction

Reactive oxygen species (ROS), spontaneously generated by metabolic enzymes, regulate cellular response as signal molecules. However, excessive ROS have cellular toxicity and cause various diseases such as cancer, brain diseases, heart diseases and autoimmune diseases by DNA denaturation, cell membrane degradation and protein degradation [1]. The cumulated data demonstrate that particulate matter (PM) has toxic effects on cardiovascular systems as well as eyes, skin and respiratory systems [2,3,4]. Many researchers have revealed through in vitro studies that PM exposure induces migratory activity dysfunction and a decrease in corneal survival through oxidative stress, inflammation and autophagy [5,6,7,8]. A study demonstrated that oxidative stress induced by both PM2.5 and PM10 collected from road dust are not different, but the cellular responses are dependent on the concentration and solubility [9]. Dry eye disease-like symptoms reported in animal studies using PM2.5 and PM10 are not largely different [5,10], and the progression of the symptoms is probably more dependent on the inflammatory response [11]. In our previous study, the adverse effects of urban particulate matter (UPM) were widely explored in the cornea, conjunctiva and retina in a time-dependent manner [12]. Our results demonstrated that long-term exposure of the eyes to UPM induces dry eye-like symptoms, inflammatory responses and deleterious structural changes in the cornea, conjunctiva and retina. Based on these studies, the cornea has become the major target for the development of a protective agent against external risks because it is constantly exposed to the environment and easily injured. Additional defects caused by UPM exposure, including tear film instability, presbyopia, allergic conjunctivitis, toxicity, oxidative stress and inflammation, have been reported in in vivo studies [13,14,15,16]. These defects are easily induced by short-term exposure to UPM, even though the exposure methods and sources of UPM are different among different studies [5,10,17,18].

Corneal abrasion is a type of injury that commonly occurs on the corneal surface and affects nearly 2% of the population annually [19]. The causes of this kind of injury vary widely, from shallow scratches from daily activities such as rubbing, makeup, applying contact lenses and dust to severe wounds such as surgery and accidents [19,20,21]. After minor injury, the corneal epithelium rapidly recovers within 1~2 days due to the migration and proliferation of epithelial cells [22,23]. However, normal wound healing can be delayed by the depth and nature of injury or metabolic disorders and result in scarring of the tissue or major corneal issues [19,21]. Furthermore, UPM can cause delayed corneal wound healing, as described above, by well-studied mechanisms [24]. However, the direct effects of UPM on the wound healing process have not been sufficiently investigated because most studies have performed only in vitro experiments. Some studies have shown enhanced wound healing using numerous molecules, but although certain therapeutic strategies have been suggested, such as increasing the migration and proliferation of epithelial cells, no agent has been successfully identified [25,26,27,28]. In addition, some agents require repeated applications due to their low bioavailability, which presents a limitation [29,30]. Thus, efforts to discover a new approach to improve delayed wound healing after exposure to disturbing factors, such as UPM, are necessary. Therefore, this study was designed to investigate the effects of UPM on corneal wound healing and find a new protective agent. Such an agent should be safe, easy to apply and have multiple functions, such as enhancing the migratory activity of epithelial cells and having antioxidative and anti-inflammatory properties. Herbal extracts can be a solution due to their safety and multiple protective functions.

*Peucedanum japonicum* Thunb. (family *Apiaceae*) is traditionally used as a vegetable in the East Asia region, but it is sometimes used as a medicine for headaches, colds, neuralgia, rheumatoid arthritis and other inflammatory diseases [31,32]. *P. japonicum* has been reported to contain coumarins, phenolic compounds, chromones, polyacetylenes, inositols and steroid glycosides [33,34]. *P. japonicum* has anti-obesity [35,36], antiplatelet [37,38], antiallergic [39,40], antioxidative [41,42] and anti-inflammatory properties [30]. Chlorogenic acid (CA) and its isomers, neochlorogenic acid (NCA) and cryptochlorogenic acid (CCA), are known as its major constituents, and their antioxidative functions have been reported [41]. Because CAs are abundant in foods, especially coffee, they have attracted a lot of attention, but they also have other well-established beneficial effects on cardiovascular, metabolic, neuronal and hepatic diseases, even cancer [43]. However, the protective effects of the CAs found in *P. japonicum* on wound healing, especially after UPM exposure, are not well studied. Therefore, the leaf extract of *P. japonicum* (PJE) was assessed for its effect on the wound healing process, and its mechanism and active molecules were investigated in this study.

## 2. Materials and Methods

### 2.1. Reagents

In this study, we used commercially available UPM (Standard Reference Material^®^ 1648a (SRM1648a), National Institute of Standards Technology, Gaithersburg, MD, USA). The detailed information (certificate of analysis) on SRM1648a can be accessed via this link (Material details of SRM1648a. Available online: https://www-s.nist.gov/srmors/view_detail.cfm?srm=1648a (accessed on 21 October 2021)).

### 2.2. Sample Preparation and HPLC Analysis

**Figure 1:**
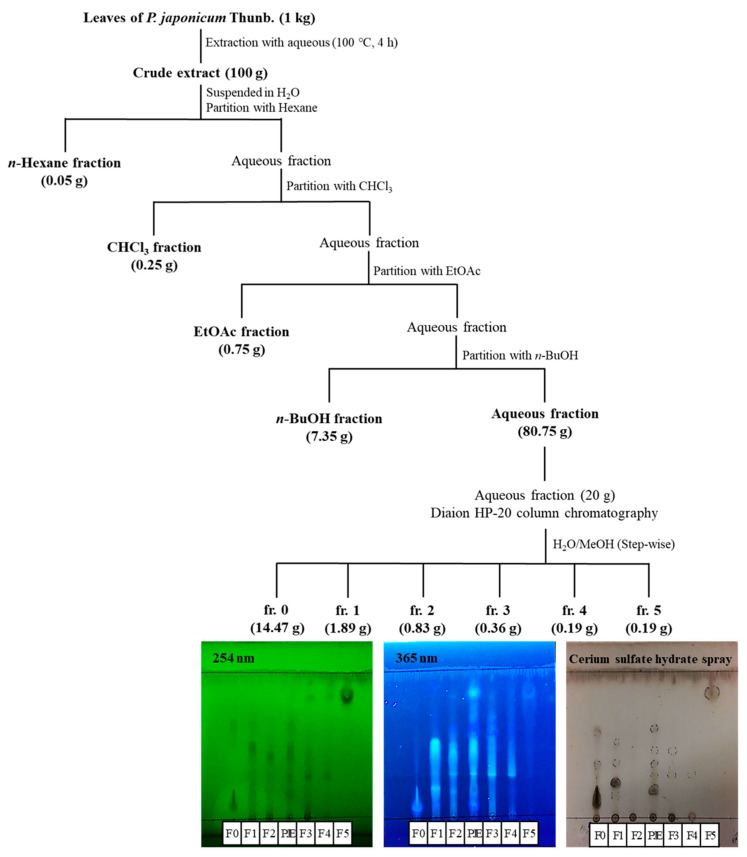
Scheme of PJE fractionation by solvent-solvent extraction and open column chromatography. TLC analysis images are also presented.

In order to ensure the validation of PJE and reproducibility of efficacy, *P. japonicum* Thunb., harvested by selecting the region (34°35′24.2″ N, 126°48′09.3″ E, Gangjin-gun, Jeollanamdo, Korea) and season (September), was used in this study. The branches and roots were removed, and only the leaf part was selected and dried in the hot air oven at 45 °C for 48 h. The dried leaves of *P. japonicum* Thunb. (1 kg) were extracted using 20 volumes of water at 100 °C for 4 h. The extracted solution was then filtered, concentrated with an evaporator under vacuum and spray dried, which gave the PJE sample. For solvent fractionation, the dried water extract (PJE, 100 g) was resuspended in water and partitioned with 𝑛-hexane (3 × 1 L), chloroform (CHCl_3_; 3 × 1 L), ethyl acetate (EtOAc; 3 × 1 L) and 𝑛-butanol (BuOH; 3 × 1 L) (Figure 1). The samples were dissolved in 50% methanol, sonicated for 20 min and then analyzed using an Agilent 1260 HPLC system (Agilent Technologies, Palo Alto, CA, USA) equipped with an Eclipse XDB-C18 column (4.6 × 250 mm, 5 μm). The temperature of the column oven was maintained at 40 °C. The mobile phase for liquid chromatography (LC) consisted of 0.1% (*v*/*v*) formic acid in water (solvent A) and 70% (*v*/*v*) acetonitrile in water (solvent B) and was applied as follows: 0–20 min, 2–12.5% (B); 20–30 min, 12.5–13% (B); 30–50 min, 13–40% (B); and 50–51 min, 40–2% (B), followed by re-equilibration with 2% (B) for 51–55 min at a flow rate of 1 mL/min and an injection volume of 10 μL. The detection wavelength was set to 330 nm. Quantitative HPLC analysis was replicated three times.

### 2.3. Open Column Chromatography and Thin-Layer Chromatography (TLC) Analysis

The H_2_O fraction (20 g) was separated with an HP-20 open column (6.5 × 52 cm; 250–850 μm particle size, IONTEC, Seongnam, Republic of Korea) and eluted with H_2_O/MeOH (a stepwise system of 10:0, 8:2, 6:4, 2:8, 0:10 *v*/*v*, each 8 L). Each obtained fraction was analyzed using the HPLC system. All fractions obtained from the purification process were spotted on silica gel for TLC analysis (silica gel 60 F254, 0.25 mm thickness; Merck) and run in a solvent system composed of *n*-BuOH/acetic acid/H_2_O (4:1:2, *v*/*v*/*v*) in a TLC chamber at room temperature. The purities of the fractionated compounds were determined by UV visualization after spraying with 1% cerium sulfate. All fractionation steps and TLC analysis images are illustrated in Figure 1.

### 2.4. HCEC Culture

The simian virus 40 (SV40)-transformed HCE cell line (RIKEN BioSource Center, Tokyo, Japan) was cultured in Dulbecco’s modified Eagle’s medium/Ham’s F-12 (DMEM/F-12) (Welgene, Daegu, Korea) supplemented with 5% fetal bovine serum, 5 μg/mL insulin, 10 ng/mL epidermal growth factor and 0.5% dimethyl sulfoxide (DMSO).

### 2.5. Cell Viability

Cell viability was evaluated with a 3-(4,5-dimethylthiazol-2-yl)-2,5-diphenyltetrazolium bromide (MTT) assay. Cells (2 × 10^4^ cells/well) were plated in 96-well plates, and after 24 h, the cells were treated with 100 μg/mL UPM and various concentrations of PJE (1–300 μg/mL), PJE fractions (0.1–30 μg/mL) or CAs (0.028–8.46 μM) for 48 h. The cells were then incubated with 0.5 mg/mL MTT solution for 2 h, and the generated formazan was dissolved in DMSO. Cell viability was measured with a microplate reader (BioTek, Winooski, VT, USA) at 540 nm.

### 2.6. Scratch Wound Healing Assay

The scratch wound healing assay was performed to assess the effects of UPM on the migration of HCECs as previously reported [12]. Briefly, cells were preincubated with 100 μg/mL UPM and 1–300 μg/mL PJE suspended in DMEM/F-12 medium without supplements for 24 h. Then, a steady line (scratch) was created using a sterile 200 μL pipette tip across the cells on the bottom of the culture dish. The culture media was replaced with fresh media with the appropriate dose of PJE, and photographs were taken at 0 and 24 h using a microscope equipped with a digital camera (Leica Microsystems, Wetzlar, Germany). The migration rate was calculated by relative comparison of the closed area at 24 h with the area at 0 h, as measured by ImageJ software (National Institutes of Health, Bethesda, MD, USA). The assay was repeated three times, and each area was measured at least three times. The PJE fractions and their major compounds, CA (Sigma-Aldrich, St. Louis, MO, USA), NCA (Sigma-Aldrich) and CCA (Sigma-Aldrich), were also assessed with the same method described above. The half maximal effective concentration (EC_50_) and maximal effective concentration (EC_max_) of each CA or CA mixture were calculated using the software program GraphPad Prism 5 version 5.01 for Windows (GraphPad, Inc., San Diego, CA, USA).

### 2.7. Real-Time Polymerase Chain Reaction (PCR)

After the wound healing assay, the cells were lysed with TRIzol reagent (Invitrogen, Carlsbad, CA, USA) to extract total RNA, and 2 μg of RNA was reverse-transcribed with M-MLV reverse transcriptase (Enzynomics, Daejeon, Korea) according to the manufacturer’s instructions. The sequences of the primers were as follows: for human superoxide dismutase type 1 (SOD1) (NM_000454.5): sense, 5-GGTGGGCCAAAGGATGAAGAG-3 and antisense, 5-CCACAAGCCAAACGACTTCC-3; for human CAT (NM_001752.4): sense, 5-TGGAGCTGGTAACCCAGTAGG-3 and antisense, 5-CCTTTGCCTTGGAGTATTTGGTA-3; for human HO1 (NM_002133.3): sense, 5-AAGACTGCGTTCCTGCTCAAC-3 and antisense, 5-AAAGCCCTACAGCAACTGTCG-3; for human glutathione peroxidase 1 (GPX1) (NM_000581.4): sense, 5-CAGTCGGTGTATGCCTTCTCG-3 and antisense, 5-GAGGGACGCCACATTCTCG-3; and for human β-actin (NM_001101.5): sense, 5-CTCACCCTGAAGTACCCCATC-3 and antisense, 5-GGATAGCACAGCCTGGATAGCA-3. PCR was performed for 45 cycles under the following conditions: denaturation at 95 °C for 20 s, annealing at 58 °C for 20 s and extension at 72 °C for 20 s using a CFX96 (Bio-Rad, Hercules, CA, USA) with RbTaq qPCR 2× PreMIX (Enzynomics, Daejeon, Korea). RNA levels were normalized to that of β-actin.

### 2.8. Animals

Five-week-old male Sprague–Dawley (S-D) rats were purchased from Samtako Animal, Inc. (Osan, Korea). All experimental procedures were conducted in accordance with the relevant guidelines for the care of experimental animals and approved by the Institutional Animal Care and Use Committee (IACUC) of Bioresources and Technology (B&Tech) Co., Ltd., Republic of Korea (B&Tech) (approval number: BT-003-2020). Animals were quarantined before the experiment and allowed to adapt to the environment for 1 week. All participating rats had normal ocular surfaces as evidenced with a stereomicroscope.

### 2.9. Animal Grouping and Dosing

UPM was dissolved in phosphate-buffered saline (PBS) to prepare a 5 mg/mL eye drop solution. The solution was stored in glass bottles at 4 °C and used within one week. The rats were randomly divided into 8 groups (*n* = 6/group), namely, the control groups (wounded right eye group—NR and non-wounded left eye group—NL), UPM-treated group (UPM), 25 mg/kg PJE-treated group (25), 50 mg/kg PJE-treated group (50), 100 mg/kg PJE-treated group (100), 200 mg/kg PJE-treated group (200) and 400 mg/kg PJE-treated group (400). The left eye of the normal group was used as the normal eye (NL), while the right eye was used as the wound control. All PJE and UPM groups received 10 μL of UPM solution as an eye drop into the right eye 4 times per day. Each dose of PJE was orally administered once a day. UPM and PJE administration began 5 days before wound generation and continued until the end of the experiment. For wound generation, all rats were anesthetized by intraperitoneal injection of a mixture of ketamine (50 mg/kg) and xylazine (5 mg/kg), and a 4 mm diameter wound was created on the right eye with an Alger brush rotating burr tool (The Alger Company Inc., Lago Vista, TX, USA). The wounded corneas were stained with fluorescein solution, and images were captured at 0, 8, 16, 24, 36, 48 and 72 h. Then, the animals were sacrificed, and the eyeballs were collected for further analysis.

### 2.10. Fluorescein Staining

Fluorescein staining, tear volume and tear break-up time (TBUT) were performed as previously described [12]. Briefly, the wounded area was stained with 2 μL of 0.5% sodium fluorescein solution, and images were captured with a funduscope with an integrated fiber optic light source equipped with a low light fluorescence camera (OcuScience, Henderson, NV, USA). The time-dependent changes in wounded area were calculated and compared with the area at 0 h, which was measured by ImageJ software (National Institutes of Health, Bethesda, MD, USA).

### 2.11. Hematoxylin and Eosin (H&E) Staining

Eyeballs were fixed for 48 h in 10% buffered formalin. Then, they were coronally bisected to separate the cornea from the retinal region. The corneas were embedded in paraffin and sliced into 4 μm thick sections. The sections were deparaffinized and rehydrated using standard techniques and stained with Mayer’s hematoxylin and 1% eosin. The stained sections were dehydrated and cleared with xylene. Mounting was performed using Canada balsam and the sections were observed under a light microscope (Nikon, Tokyo, Japan). The immune cells’ infiltration in the central and lateral cornea was calculated by measuring densities of nuclei. Covered area by re-epithelialization compared to whole corneal surface area and cell attached area in inner cornea were also calculated. All histological changes were quantified by ImageJ software (National Institutes of Health, Bethesda, MD, USA).

### 2.12. Terminal Deoxynucleotidyl Transferase dUTP Nick End Labeling (TUNEL) Staining

The number of apoptotic cells was determined with an In Situ Cell Death Detection kit (Sigma, St Louis, MO, USA) following the manufacturer’s instruction. Tissue sections were stained with DAPI containing mounting medium to visualize all nuclei. The apoptotic cells were counted under a fluorescence microscope, and their number in three different regions of each section were used for analysis. Densities of the apoptotic cells were analyzed by ImageJ software (National Institutes of Health, Bethesda, MD, USA).

### 2.13. Immunohistochemical Staining

The interleukin-6 (IL-6) in the tissue was determined by immunohistochemical staining with the anti-IL6 antibody (Abcam, Cambridge, MA, USA) and a detection kit (Dako, Carpinteria, CA, USA) including a secondary antibody. All procedures were performed according to the manufacturers’ recommendations. Nuclei were visualized by hematoxylin counter-staining. The stained sections were observed under a light microscope (Nikon, Tokyo, Japan), and images were obtained. Densities of the IL-6 were analyzed by ImageJ software (National Institutes of Health, Bethesda, MD, USA).

### 2.14. Statistical Analysis

All quantitative results were presented as the mean ± standard deviation (SD).

Statistical significances were tested using Student’s *t*-test or two-way analysis of variance (ANOVA) with GraphPad Prism 5 version 5.01 for Windows (GraphPad, Inc., San Diego, CA, USA), and the *p* values under 0.05 were marked using indicators.

## 3. Results

### 3.1. HPLC Analysis of the PJE

**Figure 2:**
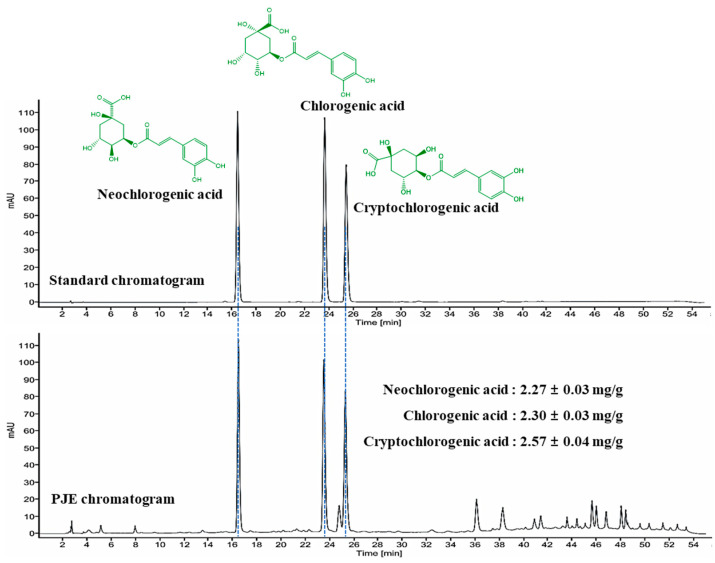
HPLC chromatograms of chlorogenic acid (CA), neochlorogenic acid (NCA) and cryptochlorogenic acid (CCA) as standard compounds and the *P. japonicum* Thunberg leaf extract (PJE).

Characterization and identification of the natural compounds in PJE were determined by HPLC. The concentrations of NCA, CA and CCA were 2.27 ± 0.03 mg/g, 2.30 ± 0.03 mg/g and 2.57 ± 0.04 mg/g, respectively. Representative chromatograms of the NCA, CA and CCA references and their corresponding peaks in the PJE are illustrated in Figure 2.

### 3.2. PJE Enhances the Migration Activity of HCECs after UPM Exposure

**Figure 3:**
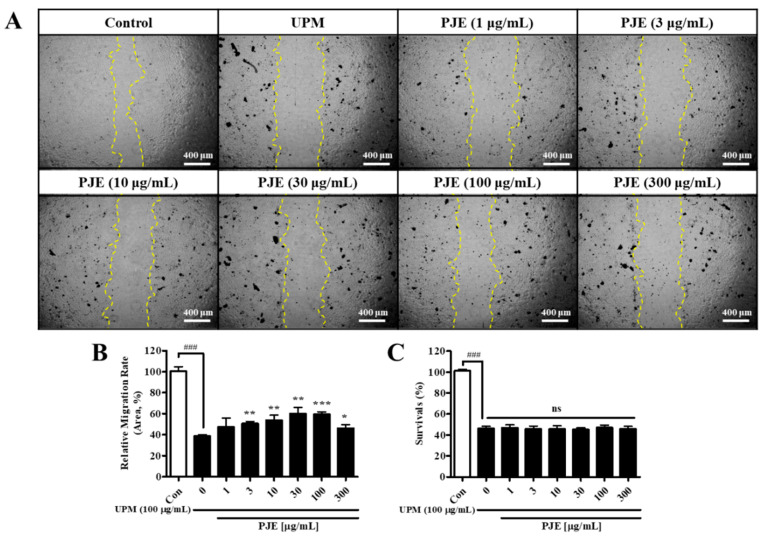
Effects of UPM and PJEs on the migration activity were assessed on the human corneal epithelial cells (HCECs). (**A**) A scratch was induced to the HCECs treated with UPM and various concentrations of PJE for 24 h. The closed area after 24 h from scratch area were calculated. The scale bars indicate 400 μm. (**B**) The relative migration rates of HCECs are expressed as the means ± SD. (**C**) The survival rates of HCECs are expressed as the means ± SD. ^###^ *p* < 0.001 compared to Con; * *p* < 0.05, ** *p* < 0.05, *** *p* < 0.001 compared to 0 μg/mL PJE; ns, not significant.

Previously, UPM decreased the migration activity and survival of HCECs [12]. Many herbal extracts were screened by scratch wound healing assay (data not shown), and PJE showed beneficial effects and was selected to confirm the preventive effects and detailed mechanisms in this study. The migration activity of HCECs was decreased to 38.7% by UPM exposure compared with normal cells. PJE treatment after UPM exposure improved the migration activity of HCECs dose-dependently until 100 μg/mL PJE, and the maximal migration reached 59.3% compared with that of normal cells, but at higher concentrations, the migration was slightly decreased, even though it was still higher than that of UPM-only exposed cells (Figure 3A,B). Cell survival was decreased by UPM exposure and was not changed by PJE treatment (Figure 3C).

### 3.3. PJE Increases Antioxidative Gene Expression in HCECs after UPM Exposure

**Figure 4:**
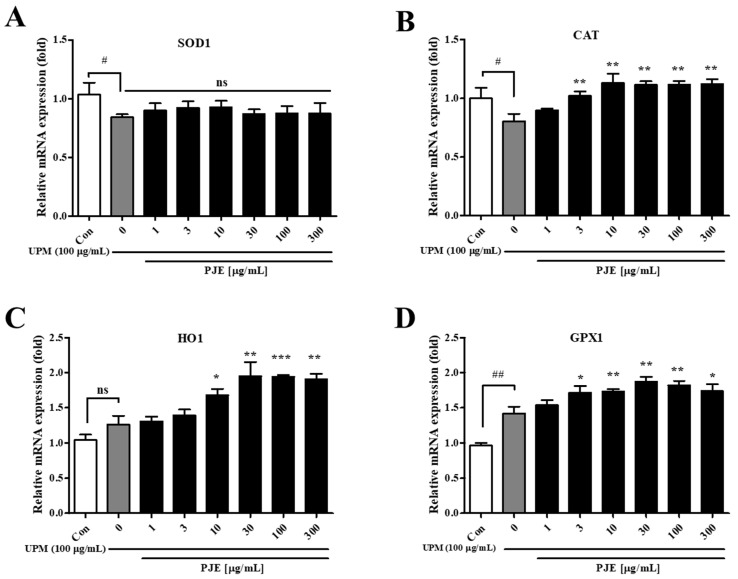
Effects of PJE on the mRNA expression of antioxidative genes in HCECs. After the scratch assay, the relative mRNA expression levels of (**A**) SOD1, (**B**) CAT, (**C**) HO1 and (**D**) GPX1 were analyzed, and the fold changes are presented compared with the Con as the means ± SD. ^#^ *p* < 0.05, ^##^ *p* < 0.01 compared to Con; * *p* < 0.05, ** *p* < 0.01, *** *p*< 0.001 compared to 0 μg/mL PJE; ns, not significant.

One of the major causes of migration activity dysfunction in HCECs is oxidative stress. The messenger RNA expression of antioxidative genes in PJE-treated cells was analyzed to confirm whether the preventive effect of PJE affected antioxidant activity. The expression of SOD1 was decreased 0.84-fold by UPM but was not changed by PJE treatment (Figure 4A). CAT expression was also decreased to 0.80-fold by UPM but increased by PJE treatment, which showed a maximal increase to 1.13-fold and the same at 10 μg/mL to 300 μg/mL PJE (Figure 4B). HO1 expression was not significantly changed by UPM exposure, but it increased 1.95-fold by PJE (Figure 4C). GPX1 expression was induced 1.42-fold by UPM and increased 1.87-fold by PJE treatment (Figure 4D). Therefore, increased antioxidative gene expression by PJE treatment after UPM exposure may contribute to the promoted migration activity of HCECs.

### 3.4. PJE Enhances Wound Healing in a Corneal Abrasion Rat Model after UPM Exposure

**Figure 5:**
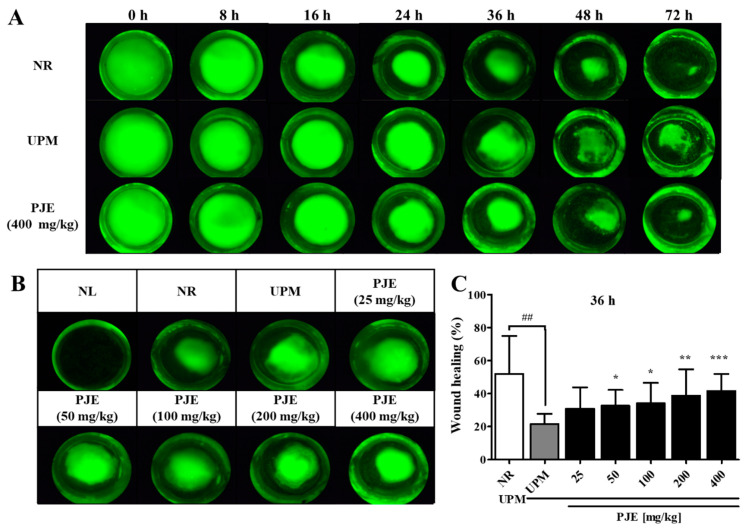
Effects of PJE in a corneal abrasion model after UPM exposure. PJE and UPM were used as pretreatments for 5 days, and 4 mm wounds were generated on the right cornea. Images were obtained at 0, 8, 16, 24, 36, 48 and 72 h by fluorescein staining. (**A**) Time course images of corneal wound healing in the normal right eye (NR, normal wound), UPM and 400 mg/kg PJE groups. (**B**) Representative images of each group at 36 h. (**C**) The wound healing areas were calculated and are presented as the means ± SD. ^##^ *p* < 0.01 compared to NR; * *p* < 0.05, ** *p* < 0.01, *** *p* < 0.001 compared to UPM.

Corneal epithelial wounds were generated on the right eyes of rats pretreated with various concentrations of PJE exposed to UPM, and their recovery rates were analyzed in a time-dependent manner. Healing of untreated corneal wounds was almost complete 72 h after wound generation (NR group), but recovery was delayed by UPM exposure (Figure 5A). The greatest difference was observed at 36 h, with a difference between the normal and UPM groups of approximately 30% (Figure 5B,C). This difference was dose-dependently decreased by PJE treatment and reached a maximum of approximately 10%. At 72 h, the area of the healed wounds in all PJE-treated groups was similar to that of the normal group, whereas the UPM group did not show sufficient recovery (Table 1).

### 3.5. Effects of PJE on Corneal Histological Changes

**Figure 6:**
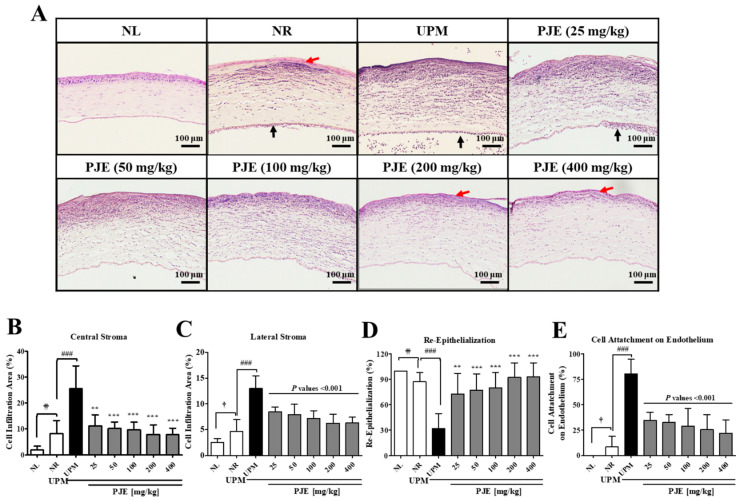
Histological changes during corneal wound healing after treatment with PJE and after UPM exposure. (**A**) Images of hematoxylin and eosin (H&E) staining of the cornea at 72 h were acquired at 200× magnification, and representative images are shown. Red arrow, re-epithelialized epithelium; black arrow, cell attachment on the endothelium. The infiltration of immune cells in (**B**) the central stroma and (**C**) the lateral stroma, (**D**) the re-epithelialization rate and (**E**) cell attachment to the endothelium were calculated, and the results are presented as the means ± SD. ^†^ *p* < 0.05, ^††^ *p* < 0.01 compared to NL; ^###^ *p* < 0.001 compared to NR; ** *p* < 0.01, *** *p* < 0.001 compared to UPM.

The histological changes in the cornea at 72 h after wound generation were detected by H&E staining. As shown in Figure 6A, the wounded normal (NR) group displayed accumulation of infiltrated inflammatory cells in the central corneal stromal region, a few immune cells were attached to the endothelial layer, and the re-epithelized cell layer was also observed. UPM exposure exacerbated the inflammatory cell infiltration and re-epithelialization during corneal recovery, while PJE treatment ameliorated these effects. Specifically, inflammatory cell infiltration was slightly increased in both the central and lateral regions of the corneas in the NR group, which was strongly increased by 3-fold after UPM exposure and dose-dependently decreased by PJE treatment (Figure 6B,C). In the NR group, the wounded areas of the cornea were almost completely covered by epithelial cells (87.3% coverage), but this coverage was severely delayed by UPM exposure (32.2%) and dose-dependently enhanced by PJE treatment (Figure 6C). The area of immune cell attachment on the endothelial layer also greatly increased to 80.1% due to UPM exposure, which was significantly decreased by PJE treatment (Figure 6E). Therefore, these data demonstrated that PJE administration to treat corneal wound healing after UPM exposure significantly inhibited inflammatory cell infiltration and enhanced re-epithelialization.

### 3.6. PJE Inhibits IL-6 Expression during Corneal Wound Healing after UPM Exposure

**Figure 7:**
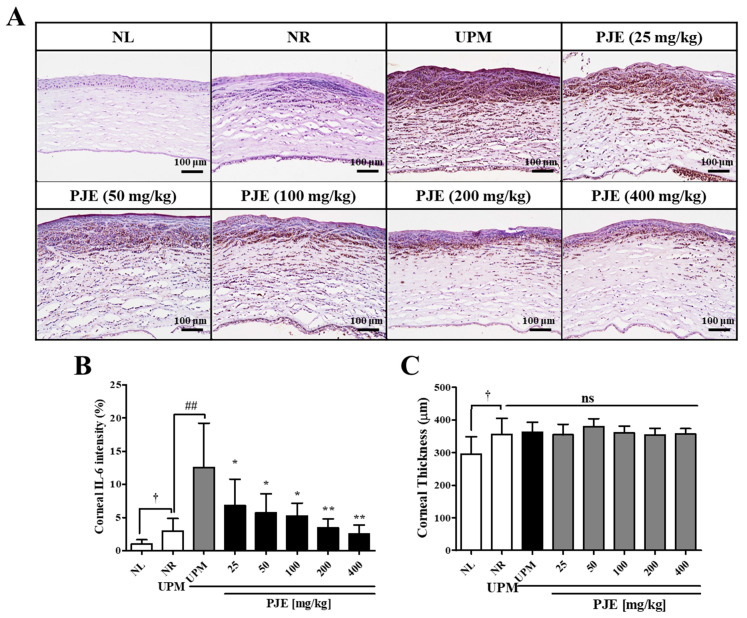
Immunohistological changes in IL-6 expression during corneal wound healing after treatment with PJE and after UPM exposure. (**A**) Immunohistochemical staining images for IL-6 in the corneas after 72 h were acquired at 200× magnification, and representative images are shown. (**B**) The intensities of the stained areas were calculated and the data are presented as the means ± SD. (**C**) The corneal thicknesses were calculated and are presented as the means ± SD. ^†^ *p* < 0.05 compared to NL; ^##^ *p* < 0.01 compared to NR; * *p* < 0.05, ** *p* < 0.01 compared to UPM; ns, not significant.

The distribution of IL-6, a major player in the immune response during corneal wound healing, was analyzed by immunohistochemical staining. As shown in Figure 7A,B, IL-6-stained regions were rarely found (2.9% of the area) in the NR group, but these regions largely increased to 12.6% after UPM exposure, which was dose-dependently decreased by PJE treatment, showing results that were close to the NR level. IL-6 expression was mainly distributed in the subsuperficial region of the stroma of the wounded corneas. Corneal thickness increased significantly due to wound generation but was unchanged after UPM exposure or PJE treatment (Figure 7C).

### 3.7. PJE Inhibits Apoptosis during Corneal Wound Healing after UPM Exposure

**Figure 8:**
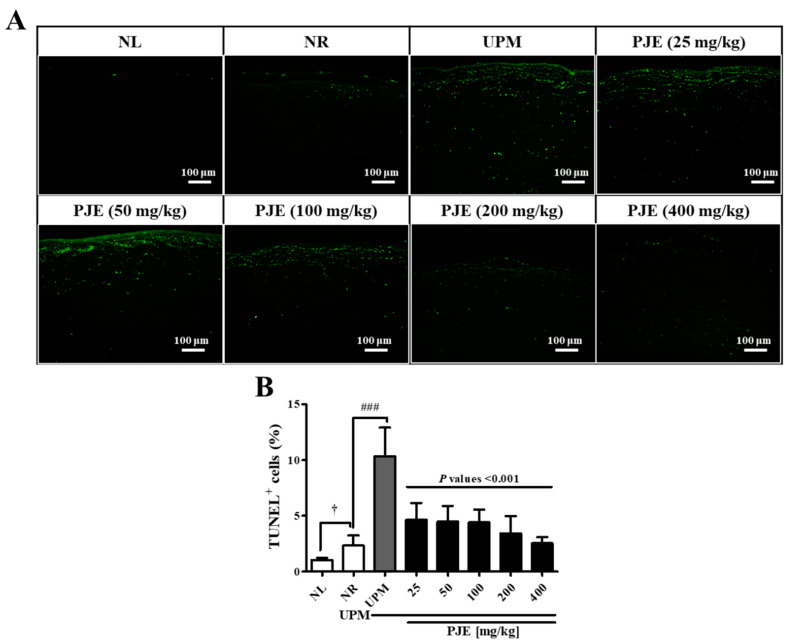
Apoptotic cells during corneal wound healing after treatment with PJE and after UPM exposure. (**A**) TUNEL staining images of the cornea at 72 h were acquired at 200× magnification, and representative images are shown. (**B**) The intensities of the apoptotic cells were calculated, and the results are presented as the means ± SD. ^†^ *p* < 0.05 compared to NL; ^###^ *p* < 0.001 compared to NR. The *p* values of all PJE groups were below 0.001 compared to UPM.

As shown in Figure 8A,B, the few apoptotic cells (2.3%) found in the NR group greatly increased to 10.4% by UPM exposure, which was significantly decreased in all PJE treatment groups, reaching the level of the NR group. Apoptotic cells were mainly distributed in the superficial region of the stroma and showed a slightly different distribution than that of IL-6.

### 3.8. Effects of PJE Solvent Fractionation on HCEC Wound Healing after UPM Exposure

**Figure 9:**
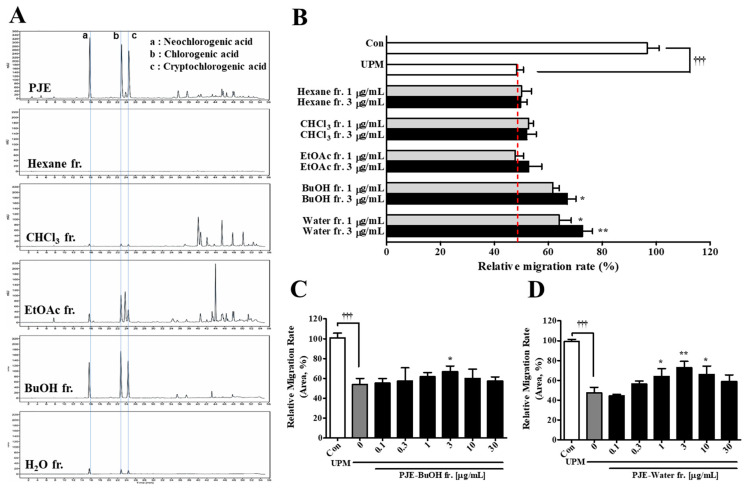
Fractions of PJE after solvent fractionations and their effects on HCEC wound healing. (**A**) HPLC chromatograms of the PJE and its fractions. (**B**) The migration rates of each fraction were calculated, and data from the concentrations of 1 μg/mL and 3 μg/mL are presented as the means ± SD. (**C**) The migration rates of the (**C**) BuOH fraction and (**D**) water fraction are presented as the means ± SD. ^†††^ *p* < 0.001 compared to Con; * *p* < 0.05, ** *p* < 0.01 compared to 0 μg/mL PJE.

PJE was separated by solvent fractionation, and each fraction and assessed for its preventive effects on wound healing after UPM exposure. The HPLC chromatograms of each fraction are shown in Figure 9A, and the peaks of the major components (CAs) were mainly found in the BuOH fraction. The chloroform, ethyl acetate and water fractions showed CA peaks (approximately 16–20 min), but many other peaks (approximately 36–52 min) were also found in the chloroform and ethyl acetate fractions; notably, the water fraction clearly showed only CA peaks. Among the fractions, both the BuOH and water fractions enhanced the migration activity of the cells (Figure 9B) without altering their survival (Supplement Figure S1A–E). Both the BuOH and water fractions showed increasing migratory activity patterns at concentrations from 0.1 μg/mL to 3 μg/mL, with a slight decrease at 30 μg/mL (Figure 9C,D). The pattern of these data presented a bell-shaped graph, which was the same as the data after PJE treatment, as shown in Figure 3B, although the water fraction showed a better effect than the BuOH fraction. To indirectly determine whether the residual peaks affected migration activity, mixtures of the BuOH and water fractions (1:2, 1:1 and 2:1) were assessed. The migration activities were enhanced by 20% after treatment with the 1:2 and 1:1 mixtures, and this activity was maintained at higher concentrations; additionally, the activity was slightly enhanced up to a concentration 3 μg/mL of the 2:1 mixture and then reduced at higher concentrations (Supplement Figure S2A–C). Therefore, the unknown components corresponding to the residual peaks in the PJE were predicted to inhibit the migratory activity at high concentrations, which was considered to be the cause of the bell-shaped curves.

### 3.9. Open Column Chromatography of the PJE Water Fractions and Their Effect on the Wound Healing of HCECs after UPM Exposure

**Figure 10:**
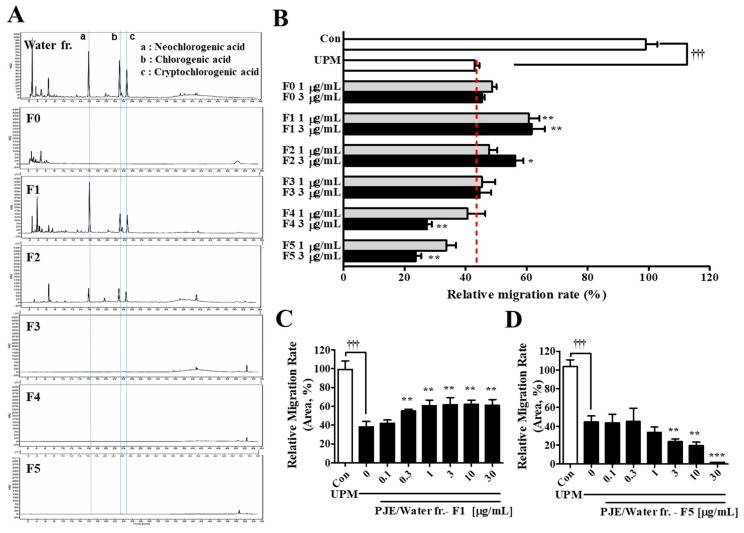
HP-20 open column chromatography of the water fraction of PJE (PJE/W) and the effects of these fractions on HCEC wound healing. (**A**) HPLC chromatograms of PJE and its fractions (F0~F5). (**B**) The migration rates of each fraction were calculated, and data from the concentrations of 1 μg/mL and 3 μg/mL are presented as the means ± SD. (**C**) The migration rates of (**C**) F1 and (**D**) F5 are presented as the means ± SD. ^†††^ *p* < 0.001 compared to Con; * *p* < 0.05, ** *p* < 0.01 *** *p* < 0.001 compared to 0 μg/mL PJE.

The water PJE fraction was separated with an HP-20 column to purify the effective components and confirm its preventive effects. The HPLC chromatograms of each fraction (F0~F5) are shown in Figure 10A. The peaks from the CAs were mainly found in F1~F2, and some residual peaks appeared in F3~F5. The migratory activities of each fraction (F0~F5) were assessed with a scratch wound healing assay, and the activity after treatment with both F1 and F2 was significantly enhanced, whereas F3~F5 at the same concentrations only slightly inhibited migration (Figure 10B). None of the fractions changed the survival rate of the cells (Supplement Figure S3A–F). F1 showed better migration activity than F2 as expected due to the greater intensity of the HPLC peaks in F1. The migration activity after F1 treatment gradually increased by 22.3% with an increase in the concentration from 0 μg/mL to 1 μg/mL, and this level was maintained at higher concentrations (Figure 10C). However, treatment with F5 led to a serial decrease in activity (Figure 10D).

### 3.10. The Preventive Effects of PJE on the Wound Healing of HCECs after UPM Exposure Were Dependent on the Three Major Compounds CA, NCA and CCA

**Figure 11:**
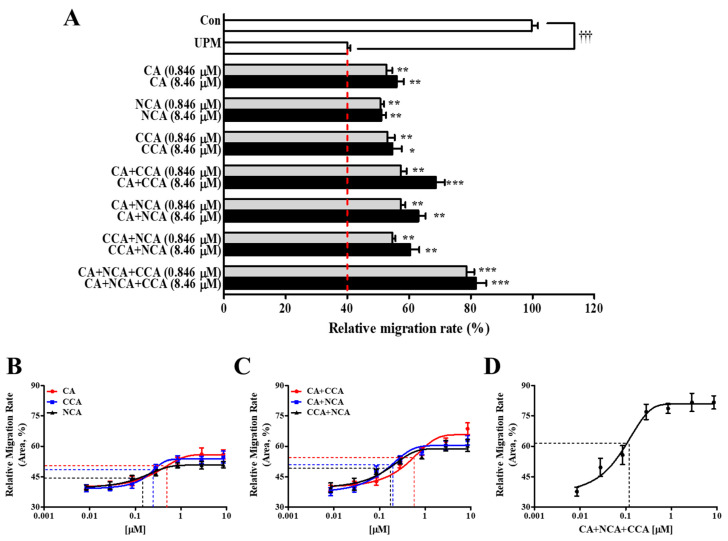
Effects of the major components of PJE on wound healing. (**A**) The migration rates of each CA and their mixtures were calculated, and data from the concentrations of 0.846 μM and 8.46 μM are presented as the means ± SD. The relative migration rates of (**B**) each CA individually, (**C**) double CA mixtures and (**D**) the triple CA mixture are presented as the means ± SD; their EC_50_ values are indicated by dotted lines. ^†††^ *p* < 0.001 compared to Con; * *p* < 0.05, ** *p* < 0.01 *** *p* < 0.001 compared to 0 μg/mL PJE.

Finally, the results of this study suggested that the preventive effects of PJE on HCEC wound healing after UPM exposure were dependent on the three major compounds CA, NCA and CCA. To confirm the effectiveness of each compound, each CA individually, each mixture of two CAs and a mixture of all three CAs were assessed for their migration activity (Figure 11A). Their concentration ranges were determined similarly to the above-tested PJE levels. All tested CA materials showed significantly increased activity, and mixtures of two or three had better responses than the single agents. None of the CAs changed cell survival (Supplement Figure S4A–C). Their EC_50_ and EC_max_ values are shown in Table 2. The EC_50_ were NCA (0.203 ± 0.040 μM), CCA (0.235 ± 0.069 μM) and CA (0.458 ± 0.170 μM), whereas the EC_max_ values were NCA (51.0%), CCA (54.7%) and CA (56.0%) (Figure 11B). These results indicate that the response occurred at low concentrations in the order of NCA, CCA and CA but showed the highest effect in the order of CA, CCA and NCA without significance. The EC_max_ values also showed a similar pattern to the EC_50_ values. The features of each CA were also displayed as a result of the EC_50_ values of each mixture of two CAs. The EC_50_ values of each mixture were CCA + NCA (0.209 ± 0.044 μM), CA + NCA (0.210 ± 0.025 μM) and CA + CCA (0.630 ± 0.062 μM), whereas the EC_max_ values were CCA + NCA (60.3%), CA + NCA (63.0%) and CA + CCA (68.7%) (Figure 11C). The mixture of three CAs showed the lowest EC_50_ (0.150 ± 0.020 μM) and the highest EC_max_ value (81.7%) (Figure 11D). Interestingly, a bell-shaped curve did not appear after CA treatment. These results could be the reason why the animal experiment showed better than expected results because the CAs synergistically enhanced the preventive effects.

## 4. Discussion

A shallow scratch on the ocular surface quickly heals through the migration and proliferation of corneal epithelial cells. However, normal wound healing can be disturbed by a deep mechanical scratch or by interrupting factors such as metabolic disorders, an allergic response, inflammation or dry eye, which can lead to adverse effects, including amblyopia, cloudiness, irritation and permanent opacification [44,45,46]. Recently, UPM has been found to be a cause of various ocular diseases, including dry eye-like disease, allergic conjunctivitis and presbyopia, because its presence has increased due to industrial development and its easy daily exposure to the eye [13,14,15]. The harmful effects of UPM on the eye have also been substantially investigated, and its cytotoxicity, immunogenicity and oxidative stimulation have been revealed; consequentially, delayed corneal wound healing after UPM exposure has also been reported in animal studies [5,6,7,8,24]. However, these data are insufficient to comprehensively understand the effects of UPM on corneal wound healing, even though the mechanisms have been suggested by various in vitro studies. Therefore, the ocular changes that occur after UPM exposure during wound healing and a screening for a preventive agent from an herbal extract were investigated in this study.

The screening of effective materials was accomplished by performing a scratch wound healing assay in HCECs after exposure to UPM, and a PJE that showed a significant effect on healing was selected as a candidate. HCEC survival was unchanged by PJE, which means that PJE enhanced the migration activity of these cells (Figure 3). It should be noted that the cells were tested without serum supplementation, which is necessary for cell proliferation, so the changes in migration activity caused by PJE were clearer.

Oxidative stress has been reported to be a major cause of epithelial dysfunction [7,8,47], and the mRNA expression levels of the key antioxidative genes SOD1, CAT, HO1 and GPX1 were quantitated by real-time PCR. CAT, HO1 and GPX1 were significantly upregulated by PJE treatment, and both HO1 and GPX1 were slightly upregulated by UPM, but SOD1 expression did not change (Figure 4). The slight increases shown after UPM treatment were thought to be due to the activation of the host defense because a similar pattern had been reported previously [48]. Enhanced antioxidative gene expression by PJE is expected to be a preventive mechanism of delayed wound healing after UPM exposure.

For animal studies, it is necessary to evaluate the effectiveness of prophylactic agents by treating the appropriate concentrations of UPM. A similar study demonstrated that PM at 5 mg/mL is suitable for analyzing molecular changes during injury [5,17]. Our previous study also showed a time-dependent change by UPM at the 5 mg/mL concentration [12]. Therefore, 5 mg/mL of UPM was applied in animal experiments. PJE was used as an oral pretreatment to rats with UPM exposure before wound generation to examine the preventive effects of PJE on corneal recovery. The restoration of larger wounds occurred in a time-dependent manner and was almost complete after 72 h, and all PJE-treated groups showed a better recovery rate than the UPM-only exposed group (Figure 5). Surprisingly, tissue analysis showed that the increased immune cell infiltration by UPM exposure was reduced by PJE, and re-epithelialization was enhanced (Figure 6). Expression of the inflammatory factor IL-6 (Figure 7) and the apoptotic cell number (Figure 8) were also decreased by PJE treatment. Normally, apoptotic cells in the corneal epithelium are involved in tissue homeostasis, and their presence can be slightly increased during wound healing to remove inflammatory cells [49,50]. In normal wound healing, a few infiltrating immune cells, such as neutrophils and macrophages, are needed to prevent infection from microbes and to enhance the recovery process by regulating marginal cells. Neutrophils have been reported to be necessary for re-epithelialization after abrasion [51,52] and to affect the recovery of the corneal nerves by vascular endothelial growth factor-A (VEGF-A) secretion [53]. Macrophages also help wound recovery by removing debris and apoptotic cells from the wound site and secreting various growth factors to induce the proliferation of epithelial cells or the differentiation of fibroblasts to myofibroblasts [49,50]. However, UPM exposure increased immune cell infiltration, and excessive inflammation was induced, as shown by the increases in IL-6 expression and apoptosis. In addition, debris clearance was delayed, as shown by the accumulation of apoptotic cells in the superficial site of the wounded cornea, whereas inflammation was still activated, as shown by the distribution of IL-6 expression in the subsuperficial site, which probably disturbed the normal recovery process.

PJE has been reported to contain numerous compounds, as described above, and three CAs showed powerful antioxidative activity [41]. Some studies also reported the anti-oxidative function of PJE by direct assay such as DPPH assay to test free radical scavenging activity. Therefore, the anti-oxidative function of PJE is one of the key properties for a delayed wound healing improvement via direct and indirect mechanisms [54,55]. Based on these data, the PJE was separated by solvent fractionation, and the effects of the fractions on the migration activity were assessed to confirm the effective molecules and their isolation conditions. None of the fractions affected cell survival, but the migration activity was improved by the BuOH and water fractions (Figure 9). However, unexpectedly, the effects of the water fraction were better than those of the BuOH fraction, although the peak sizes of the CAs in the BuOH fraction were larger. Experiments using mixtures of the BuOH and water fractions at various ratios were performed to determine whether the non-CA peaks may indirectly function as inhibitors of migration. It was found that as the ratio of the BuOH fraction increased, a reduced effect on migration activity was produced; therefore, the effects of these residual peaks were considered in the following experiments.

The water fraction showed the best effect on cell migration, but these effects were reduced at higher concentrations because small amounts of the compounds that formed the residual peaks were probably still included. Therefore, the water fraction was separated by HP-20 open column chromatography, and each fraction was assessed for its effect on migration (Figure 10). The CA peaks appeared in F1, which efficiently improved migration activity that did not decline after treatment with higher concentrations, whereas increasing concentrations of F3~F5 led to serial decreases in activity. These results indicate that some unknown inhibitory compounds are included in the PJE and that the CAs were confirmed to be the effective compounds. Finally, the effects on the migration of each CA alone or mixtures of two or three CAs were assessed to determine if each CA acted differently or whether mixtures could enhance the effects synergistically. Each CA showed slightly different EC_50_ and EC_max_ values, and the mixture of all three showed the best effect on migration. Based on these results, a mixture of all three CAs would be more effective for corneal wound healing, and the inhibitory effects of the unknown components of the PJE found through the in vitro experiments probably disappeared during metabolism. Studies in various fields have focused on CA, which is abundant in foods (especially coffee), for a long time. Many beneficial properties of CA have been reported, such as its abilities to lower the risk of metabolic syndromes and chronic diseases [56]. In addition to the beneficial effects of CA on metabolic syndromes [57], CA is also active against hepatic steatosis [58], neurodegenerative diseases [59], cardiovascular diseases [60] and cancer [61]. The antioxidative function of CA is a representative property, and it promotes diabetic wound recovery [62]. The detailed mechanisms of CA on corneal wound healing after UPM exposure will be further studied considering the relationships between each type of cell.

Herein, the preventive effects of PJE on corneal wound healing after UPM exposure were well studied through its antioxidative and anti-inflammatory properties, and the effective components were clearly defined. However, limitations of this study also exist; for example, UPM exposure from an eye drop solution is not realistic, although most similar studies have used this method; the residual peaks that inhibited migration were not identified; and the infiltrating cell types and how they interacted with other cells were not defined. Therefore, although the detailed mechanisms of CAs as preventive agents for corneal wound healing after UPM exposure focused on epithelial cells, fibroblasts and keratocyte inflammatory cells and their relationship, the molecular mechanisms should be further studied.

## 5. Conclusions

In the present study, UPM influenced corneal wound healing by causing inflammation, oxidative stress and apoptosis. PJE was selected through a screening of herbal extracts to promote the recovery rate of wounded corneas and was found to efficiently improve the wound healing and migration activity of corneal epithelial cells. The major components were defined as CA, NCA and CCA, which were effective individually on the migration activity, and they synergistically enhanced this effect. These data provide valuable information for the application of PJE to treat corneal injury accompanied by inflammation and oxidative stress.

## Figures and Tables

**Table 1:** Time course observations of the changes in corneal wound healing areas after PJE treatment and UPM exposure.

		0 h		8 h		16 h		24 h		36 h		48 h		72 h	
		Average	*p* Value	Average	*p* Value	Average	*p* Value	Average	*p* Value	Average	*p* Value	Average	*p* Value	Average	*p* Value
	NR	2.7		5.8		24.2		34.5		52.0		55.9		81.5	
	UPM	1.4	0.352	4.9	0.623	9.4	0.173	19.8	0.252	21.6	0.009 ^##^	27.1	0.019 ^#^	62.1	0.004 ^##^
PJE (mg/kg)	25	1.7	0.775	6.9	0.232	12.4	0.238	25.6	0.342	30.9	0.132	35.1	0.278	74.6	0.005 **
	50	3.3	0.111	2.1	0.068	9.4	0.999	21.6	0.793	32.6	0.031 *	41.1	0.112	80.2	0.005 **
	100	2.7	0.366	4.2	0.667	10.5	0.696	26.8	0.314	34.1	0.047 *	44.5	0.027 *	79.6	0.009 **
	200	2.5	0.465	5.5	0.770	12.6	0.350	31.1	0.103	38.7	0.028 *	48.1	0.004 **	80.4	0.003 **
	400	1.4	0.954	5.2	0.821	12.8	0.104	27.7	0.204	41.6	0.001 **	51.6	0.006 **	79.2	<0.001 ***

The mean and *p* values are presented. ^#^ *p* < 0.05, ^##^ *p* < 0.01 compared to NR; * *p* < 0.05, ** *p* < 0.01, *** *p* < 0.001 compared to UPM.

**Table 2:** The EC_50_ and EC_max_ values of each CA and their mixtures on wound healing.

	EC_50_	EC_max_
	[μM]	
CA	0.458 ± 0.170	2.637 ± 0.615
CCA	0.235 ± 0.069 ^##^	0.897 ± 0.232 ***^,###^
NCA	0.203 ± 0.040 ^##^	0.785 ± 0.105 ***^,###^
CA + CCA	0.630 ± 0.062	3.103 ± 0.337
CA + NCA	0.210 ± 0.025 ^##^	0.930 ± 0.057 ***^,###^
CCA + NCA	0.209 ± 0.044 ^##^	0.975 ± 0.086 ***^,###^
CA + CCA + NCA	0.150 ± 0.020 *^,###^	0.749 ± 0.073 ***^,###^

The values are presented as the means ± SD. ^##^ *p* < 0.01, ^###^ *p* < 0.001 compared to CA + CCA; * *p* < 0.05, *** *p* < 0.001 compared to CA.

## Data Availability

The data presented in this study are available in article and supplementary material.

## Supplementary Materials

The following are available online at https://www.mdpi.com/article/10.3390/antiox10111717/s1, Figure S1: Effects of PJE solvent fractionation on HCEC survival, Figure S2: Effects of PJE fraction mixtures (BuOH and water fractions) on HCEC wound healing, Figure S3: Effects of the PJE/Water fractions after HP-20 open column chromatography on the survival of HCECs, Figure S4: Effects of the CA isomers on the survival of HCECs.
