# Supplementary material for: Peucedanum japonicum Thunberg and Its Active Components Mitigate Oxidative Stress, Inflammation and Apoptosis after Urban Particulate Matter-Induced Ocular Surface Damage
Source: Antioxidants (Basel). 2021 Oct 28;10(11):1717. doi: 10.3390/antiox10111717 (PMC8614870; doi:10.3390/antiox10111717)
Supplement: Supplementary file 1 [file antioxidants-10-01717-s001.zip › antioxidants-1398803-supplementary.pdf]

Supplement data

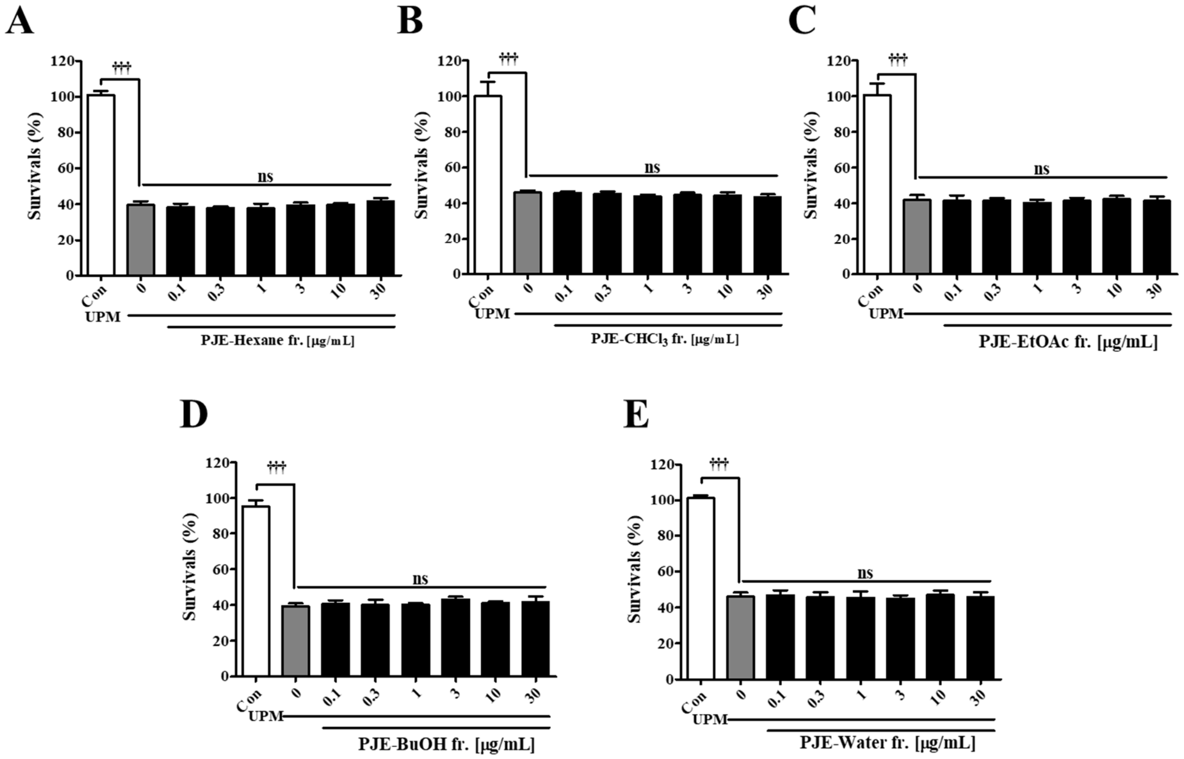

Supplement Figure S1. Effects of PJE solvent fractionation on HCEC survival. Cell survival rates after treatment with the (**A**) hexane fraction, (**B**) chloroform fraction, (**C**) ethyl acetate fraction, (**D**) butanol fraction, and (**E**) water fraction are presented as the means ± SD. $^{†††}p <$ 0.001 compared to Con; ns, not significant.

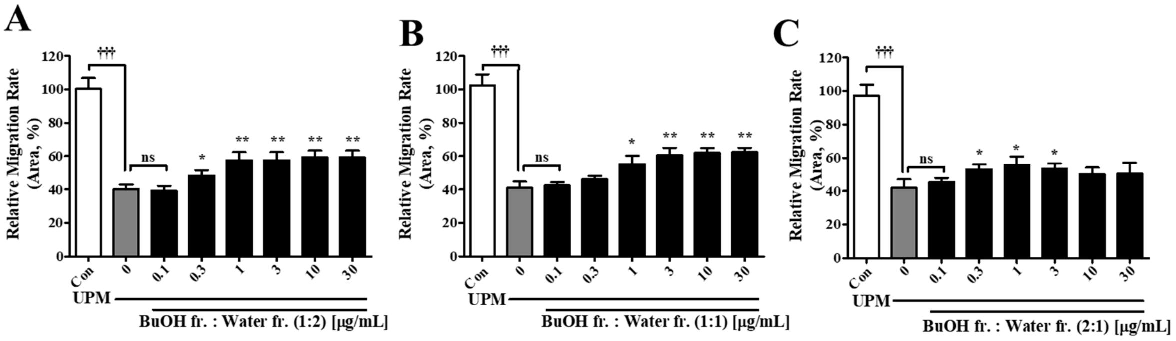

Supplement Figure S2. Effects of PJE fraction mixtures (BuOH and water fractions) on HCEC wound healing. The migration activities after treatment with the BuOH and water fraction mixtures at ratios of (**A**) 1:2, (**B**) 1:1, and (**C**) 2:1 are presented as the means ± SD. $^{†††}p < 0.001$ compared to Con; $^{*}p < 0.05$, $^{**}p < 0.01$ compared to 0 μg/mL PJE; ns, not significant.

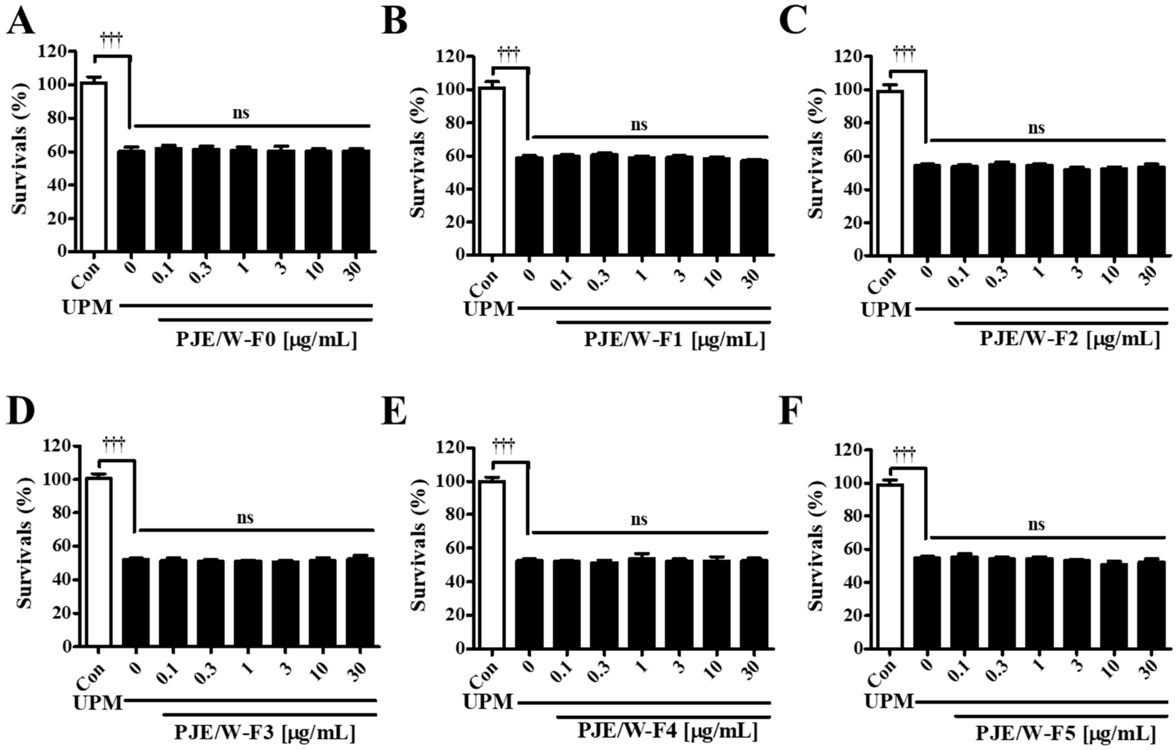

Supplement Figure S3. Effects of the PJE/Water fractions after HP-20 open column chromatography on the survival of HCECs. Cell survival rates after treatment with (**A**) F0, (**B**) F1, (**C**) F2, (**D**) F3, (**E**) F4, and (F) F5 are presented as the means ± SD. $^{†††}p < 0.001$ compared to Con; ns, not significant.

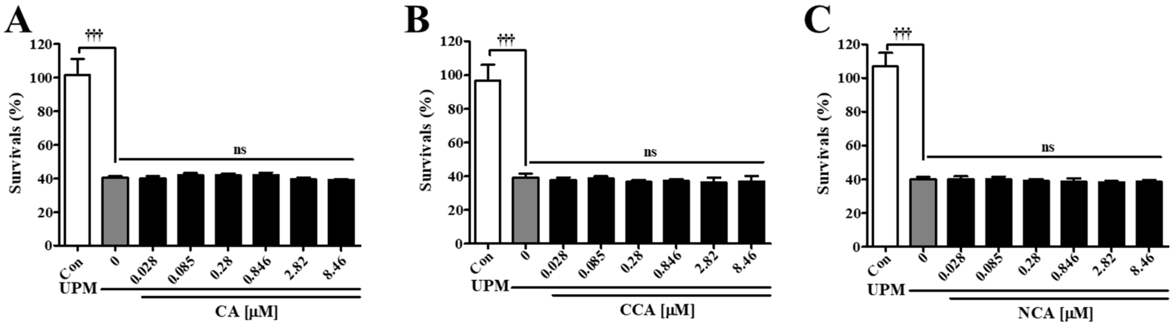

Supplement Figure S4. Effects of the CA isomers on the survival of HCECs. Cell survival rates after treatment with (**A**) CA, (**B**) CCA, and (**C**) NCA are presented as the means ± SD. ††† $p < 0.001$ compared to Con; ns, not significant.
